# Self-Assembly-Directed Exciton Diffusion in Solution-Processable Metalloporphyrin Thin Films

**DOI:** 10.3390/molecules27010035

**Published:** 2021-12-22

**Authors:** Abhishek Shibu, Camilla Middleton, Carly O. Kwiatkowski, Meesha Kaushal, Jonathan H. Gillen, Michael G. Walter

**Affiliations:** Department of Chemistry, University of North Carolina at Charlotte, Charlotte, NC 28223-0001, USA; ashibu@uncc.edu (A.S.); c.middleton01@outlook.com (C.M.); ckwiatk2@uncc.edu (C.O.K.); meesha.kaushal@gmail.com (M.K.); jgillen1@uncc.edu (J.H.G.)

**Keywords:** metalloporphyrin, self-assembly, exciton diffusion

## Abstract

The study of excited-state energy diffusion has had an important impact in the development and optimization of organic electronics. For instance, optimizing excited-state energy migration in the photoactive layer in an organic solar cell device has been shown to yield efficient solar energy conversion. Despite the crucial role that energy migration plays in molecular electronic device physics, there is still a great deal to be explored to establish how molecular orientation impacts energy diffusion mechanisms. In this work, we have synthesized a new library of solution-processable, Zn (alkoxycarbonyl)phenylporphyrins containing butyl (ZnTCB_4_PP), hexyl (ZnTCH_4_PP), 2-ethylhexyl (ZnTCEH_4_PP), and octyl (ZnTCO_4_PP) alkoxycarbonyl groups. We establish that, by varying the length of the peripheral alkyl chains on the metalloporphyrin macrocycle, preferential orientation and molecular self-assembly is observed in solution-processed thin films. The resultant arrangement of molecules consequently affects the electronic and photophysical characteristics of the metalloporphyrin thin films. The various molecular arrangements in the porphyrin thin films and their resultant impact were determined using UV-Vis absorption spectroscopy, steady-state and time-resolved fluorescence emission lifetimes, and X-ray diffraction in thin films. The films were doped with C_60_ quencher molecules and the change in fluorescence was measured to derive a relative quenching efficiency. Using emission decay, relative quenching efficiency, and dopant volume fraction as input, insights on exciton diffusion coefficient and exciton diffusion lengths were obtained from a Monte Carlo simulation. The octyl derivative (ZnTCO_4_PP) showed the strongest relative fluorescence quenching and, therefore, the highest exciton diffusion coefficient (5.29 × 10^−3^ cm^2^ s^−1^) and longest exciton diffusion length (~81 nm). The octyl derivative also showed the strongest out-of-plane stacking among the metalloporphyrins studied. This work demonstrates how molecular self-assembly can be used to modulate and direct exciton diffusion in solution-processable metalloporphyrin thin films engineered for optoelectronic and photonic applications.

## 1. Introduction

After billions of years of evolution, nature has optimized the process of photosynthesis through which living organisms can harvest sunlight for sustenance [1,2]. When sunlight is absorbed by a chromophore or an array of chromophores, the energy captured is transferred to reaction centers for dissociation of charges [3,4]. This charge separation leads to the storage of chemical energy which is later used for metabolic activities [5]. These photophysical processes of absorption of light, migration of energy, and separation of charges have influenced the field of bio-inspired solar cell fabrication tremendously [6,7,8]. Persuaded by nature’s efficiency, molecular engineers have been studying structural analogues of chlorophyll (porphyrins) which show attractive photophysical properties [9,10,11]. Porphyrins are tetrapyrrole-based aromatic macrocycles containing fully conjugated 18 π-electrons. Porphyrins have been used as linkers in metal and covalent organic frameworks and for a wide variety of applications, such as photovoltaics, photocatalysis, and energy storage [12,13,14]. Porphyrins have also been explored for biomedical applications, photodynamic therapy, sensing and spintronics [15,16,17,18]. Despite the numerous photonic applications utilizing porphyrin materials, our understanding of energy migration in these synthetic materials is still relatively limited. Considering that energy migration is a pivotal process in organic photovoltaic devices and related photonic applications, we believe a more thorough understanding of these molecular processes will lead to more efficient porphyrin-based devices.

Our earlier studies demonstrated how varying peripheral alkyl groups on free-base (alkoxycarbonyl)phenylporphyrins would affect molecular assembly and energy diffusion in solution-cast thin films [19]. We established that peripheral alkyl groups govern molecular organization and reported exciton diffusion lengths of up to 25 nm. This was one of the longest exciton diffusion lengths reported among free-base, tetraphenyl porphyrin thin films. However, in nature, porphyrins have a metal atom in the core of the macrocycle. The metal atom flattens the molecule, facilitating higher excitonic coupling in the light-harvesting antenna assemblies [20]. It also acts as an additional binding site for neighboring molecules which yield well-defined nanostructures, and preferential transition dipole stacking which is highly desired for excited-state energy diffusion in organic thin films [21,22,23]. There is, however, an implied trade-off because the metal atom would assist in intersystem crossing of excited electrons via spin-orbit coupling, resulting in higher triplet state transitions and shortened singlet emission decay lifetime [24]. This tension makes the question of molecular organization and energy diffusion in metalloporphyrins interesting and one worth exploring.

In this study, we synthesized (alkoxycarbonyl)phenyl zinc metalloporphyrins and developed techniques for depositing highly-uniform, solution-processable thin films. UV-Vis absorption, fluorescence spectroscopy, and X-ray diffractometry were used to study the molecular organization in the thin porphyrin films. We found that out-of-plane ordering of transition dipoles increases as the linear alkyl chain length increases. We also determined that metalloporphyrins with branched peripheral alkyl chains assemble in-plane exclusively. The effect of peripheral alkyl groups on the photoluminescence of metalloporphyrin thin films was established by obtaining average singlet emission decay lifetimes in thin films. The thin films were doped with C_60_ to determine relative fluorescence quenching efficiency. We utilized the quenching efficiency and fluorescent decay lifetimes in a Monte Carlo simulation to establish the exciton diffusion coefficients and diffusion lengths of the metalloporphyrins in thin films. We report exciton diffusion lengths as high as 80.67 nm, and one of the longest among similar kinds of dye molecules [25,26]. The high transition dipole ordering and long exciton diffusion lengths in these self-assembled thin films make them highly desirable candidates for photonic and optoelectronic device fabrication.

## 2. Results

### 2.1. Zn Porphyrin Thin-Film and Solution UV-Vis Spectra

**Figure 1:**
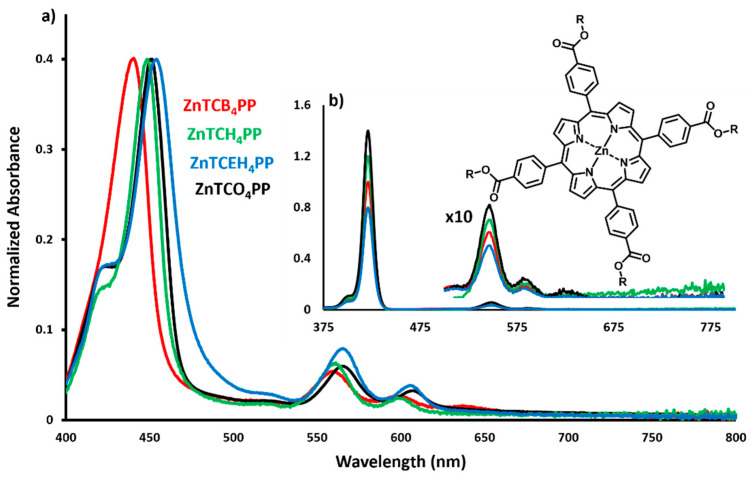
Normalized absorption spectra of (**a**) spin-cast thin films and; (**b**) solution of butyl derivative—ZnTCB_4_PP (**-**), hexyl derivative—ZnTCH_4_PP (**-**), 2-ethylhexyl derivative—ZnTCEH_4_PP (**-**) and octyl derivative—ZnTCO_4_PP (**-**). Inset: molecular structure of Zn-5,10,15,20-tetrakis(4-carboxyphenyl)porphyrin.

The UV-Vis absorption properties of Zn porphyrins in solution and thin films are measured and compared in Figure 1.

In solution, the metalloporphyrins show identical absorption characteristics with a strong Soret band at 422 nm, representing strong S_0_–S_2_ transition. The strong presence of the fourth and third Q bands can be seen at 550 nm and 588 nm, respectively. Varying the alkoxycarbonyl substituents does not alter the absorption spectra of these derivatives in solution.

The spin-cast films, however, exhibit a distinct red shift in the absorption spectra compared to their solution spectra. ZnTCB_4_PP is red-shifted the least with the Soret band at 442 nm. Increasing the length of the peripheral alkyl chain resulted in a further red shift with ZnTCH_4_PP and ZnTCO_4_PP, now showing Soret bands at 452 and 454 nm. The branched alkyl chain derivative has the most red-shifted Soret band at 455 nm. The ZnTCEH_4_PP thin film also showed the highest S_0_–S_1_ with the most prominent Q_4_ band among the four derivatives. The shifts and broadening of the Soret peaks could be attributed to various alkyl chain-dependent orientations that the Zn porphyrin molecules assemble in and the resultant π–π macrocyclic interactions [19,27,28]. The shift in spectra suggests that the peripheral alkyl chains modulate molecular transition dipole orientation and alignment in thin films and play a strong role in the electronic transitions of these materials. We observed a red shift (~25 nm) in the thin films of Zn porphyrins when compared to their free-base counterparts, which is also observed in solution. The addition of the Zn (II) ion in the porphyrin macrocycle increases the planarity of the molecule, which can facilitate intermolecular stacking in thin films. These structural changes also add a higher possibility of strong excitonic coupling of transition dipoles, leading to enhanced excited-state energy diffusion in thin films.

**Figure 2:**
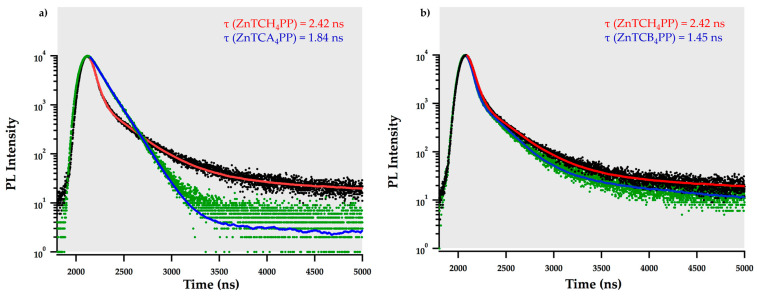
Singlet emission decay lifetime comparison of (**a**) thin film of ZnTCH_4_PP (**-**) and Zn-5,10,15,20-tetrakis(4-carboxyphenyl)porphyrin solution (**-**) and (**b**) thin films of ZnTCB_4_PP (**-**) and ZnTCH_4_PP (**-**).

The peripheral alkyl chain substituents affect the singlet emission lifetime decay as well. In Figure 2, the singlet emission decay lifetime of solution and thin films are presented below:

The singlet emission decay lifetime for all the metalloporphyrin derivatives in solution is 1.87 ± 0.03 ns and has a monoexponential fit, as shown in Figure S1. Similar to the absorption spectra, the peripheral alkyl chain substituents have no contribution to the electronic transition properties of the metalloporphyrins in solution. However, in solution-cast thin films, the emission lifetimes vary. The tri-exponential average decay lifetimes of thin films are provided in Table 1. Interestingly, ZnTCH_4_PP, ZnTCEH_4_PP, and ZnTCO_4_PP have a longer average singlet emission decay lifetime in thin films than in solution, Figure 2a. In solution, the Zn (II) ion facilitates higher triplet state transition upon interaction with the solvent molecules. Aggregation in solution could also lead to shortening of decay lifetimes. In thin films, however, the alkyl chains act as molecular spacers, preventing macrocyclic interactions with neighboring molecules. This could lead to longer decay lifetimes in thin films than in solution. The peripheral alkyl groups influence molecular ordering and self-assembly in thin films which affect the singlet decay lifetimes, as seen in Figure 2b. In the case of linear alkyl chain substituents, the emission is short-lived for smaller alkyl chains (ZnTCB_4_PP) and increases with chain length. However, after a threshold limit, the lifetimes even reduced upon the addition of futher carbon atoms on the alkyl chain. ZnTCH_4_PP has the longest decay lifetime in thin films, closely followed by ZnTCO_4_PP. The branching in the alkyl chain likely causes the molecules to assemble in an unfavorable arrangement, yielding short fluorescence decay lifetimes. The singlet emission decay lifetimes for all the materials are significantly shorter than their free-base counterparts. This is due to the heavy atom effect induced by the addition of the Zn (II) ion in the macrocycle core.

### 2.2. Exciton Diffusion Coefficient (D) and Diffusion Lengths (L_D_)

In organic semiconductors, exciton diffusion is often described by sequential Förster resonance energy transfer [29,30]. Excitons migrate randomly (diffuse) through the excited states of molecules in thin film akin to hopping. The Förster theory indicates that the rate of energy transfer is a function of distance between two weakly coupled molecular dipoles and the radius of energy transfer (*R*_o_), as shown in Equations (1) and (2):

(1) $$k_{F}=\frac{1}{\tau }{\left(\frac{Ro}{d}\right)}^{6}$$

(2) $$R_{o}=\frac{9\eta_{\mathrm{PL}}k^{2}}{128\pi^{5}n^{4}}\int \;\lambda^{4}F_{D}\left(\lambda \right)\sigma_{A}\left(\lambda \right)d\lambda$$

The radius of energy transfer (*R*_o_) is directly proportional to the orientation of molecular dipole (*k*), PL efficiency (*η*_PL_), and spectral overlap integral, where *λ* is the wavelength, *F*_D_ is the normalized fluorescence, and *σ*_A_ is the absorption cross section. Since molecular dipoles play a crucial role in excited-state energy transfer in organic thin films, engineering their orientation and strength has become a priority in materials designed for optoelectronic applications. Monte Carlo diffusion computational simulation was used to determine exciton diffusion coefficient and diffusion lengths in the metalloporphyrin thin films [31]. This simulation method has been used previously for similar molecules, conjugated polymers, and organic dyes [32,33,34,35,36]. To study steady-state quenching in thin films, C_60_ was doped in the metalloporphyrin solution in volume fraction 0.06% and 0.2% [37]. The diffusion model simulates a cube of edge length of 25 nm and calculates the hopsize before an exciton will collide with a quencher. The model requires singlet lifetime decay, dopant volume fraction, and relative quenching efficiency, which is calculated as shown in Equation (3) for input:

(3) $$Q=1-\frac{\int \;PL_{Blend}\;d\lambda }{\int \;PL_{Pristine}\;d\lambda }$$

The hopping simulation repeats with different hopsizes until it converges with the input parameters. The final resultant exciton hopsize (*s*) is then used to calculate the exciton diffusion coefficient (*D*) and exciton diffusion length (*L*_D_), as shown in Equations (4) and (5).

(4) $$D=\frac{ds^{2}}{6dt}$$

(5) $$L_{D}=\sqrt{aD\tau }$$

**Figure 3:**
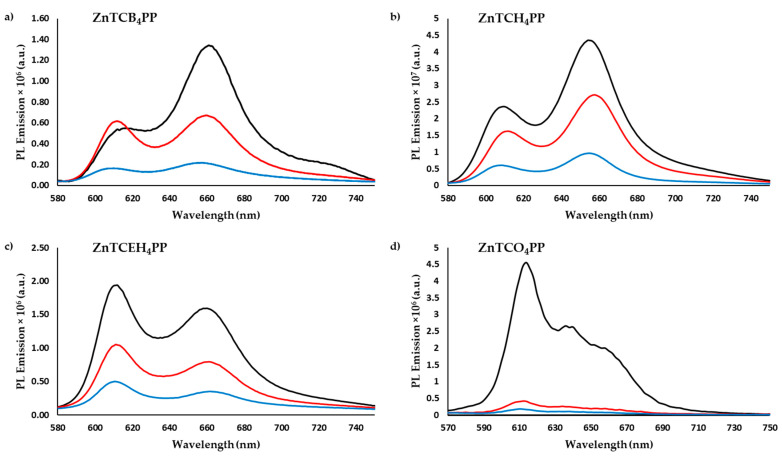
Photoluminescence emission spectra of spin-cast metalloporphyrins thin films of (**a**) butyl derivative—ZnTCB_4_PP, (**b**) hexyl derivative—ZnTCH_4_PP, (**c**) 2-ethylhexyl derivative—ZnTCEH_4_PP and (**d**) octyl derivative—ZnTCO_4_PP. Each plot includes spectra of pristine films (**-**), films doped with v_frac_ 0.06% (**-**) and films doped with v_frac_ 0.2% (**-**).

Figure 3 depicts the photoluminescence emission spectra of pristine films and films doped with a volume fraction of 0.06% and 0.2% C_60_ quencher molecules.

Doping the films with minute amounts of C_60_ quenches the emission of the films since the exciton dissociates when it encounters the electron acceptor molecule at the porphyrin–C_60_ interface. As the dopant concentration increases from *v*_frac_ 0.06% to 0.2%, the emission intensity further quenches and exciton diffusion lengths shorten. This is expected as there are now more C_60_ quencher molecules in the film. However, it should be noted that the addition of C_60_ to the porphyrin thin film does not affect their optical absorption characteristics (Figure S2). Quenching efficiencies for all the derivatives are provided in Table 1.

When calculating exciton diffusion length using Equation (5), it is evident that exciton diffusion can be modulated by tuning peripheral alkyl substituents which influences molecular packing and transition dipole orientation in thin films. The longest exciton diffusion length is observed for ZnTCO_4_PP, measuring at an astounding 80.67 nm. This is one of the highest exciton diffusion lengths observed in similar metalloporphyrin thin films and longer than the exciton diffusion length of its free-base counterpart by over three folds [19]. ZnTCO_4_PP also has an exciton diffusion coefficient which is one order above the other derivatives. The 2-ethylhexyl derivative (ZnTCEH_4_PP) has an exciton diffusion length of 13 nm and an exciton diffusion coefficient of 1.68 × 10^−4^ cm^2^ s^−1^. The butyl and hexyl derivatives have similar steady state quenching efficiency and diffusion coefficient of around 1 × 10^−4^ cm^2^ s^−1^. However, the highest diffusion length for ZnTCH_4_PP was measured at 12.26 nm, compared to 9.8 nm for ZnTCB_4_PP. This is because ZnTCH_4_PP has a higher singlet decay lifetime. Overall, ZnTCO_4_PP has the highest diffusion coefficient and diffusion length among the four derivatives. Even though the diffusion coefficient is comparable with some of the earlier reported studies, the diffusion length is certainly one of the highest among alkylated metalloporphyrins spin-cast thin films [38,39,40,41].

### 2.3. Porphyrin Thin-Film XRD Patterns

**Figure 4:**
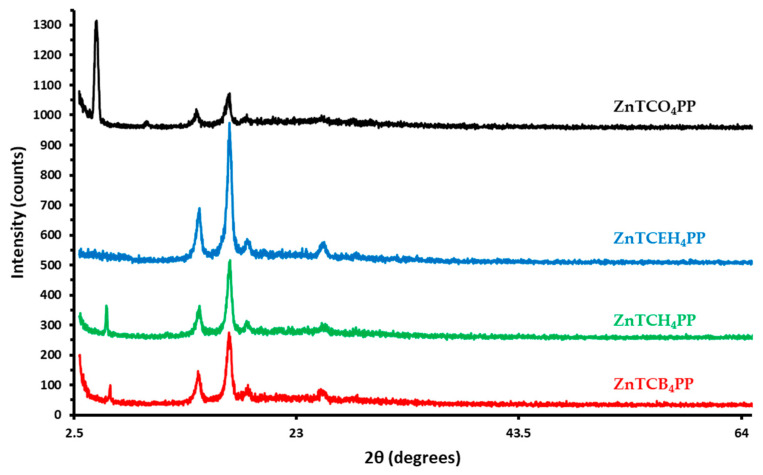
X-ray diffraction patterns of solution-cast thin films of ZnTCB_4_PP (**-**), ZnTCH_4_PP (**-**), ZnTCEH_4_PP (**-**), and ZnTCO_4_PP (**-**).

Additional molecular organization and thin film structural data were acquired from X-ray diffraction studies, as shown in Figure 4.

The presence of multiple sharp peaks (2θ = 14°, 16.85°, 18.50°, 25.45°) establish a strong molecular ordering of metalloporphyrin thin films. The diffraction peaks appearing near 2θ = 5.5° are indicative of out-of-plane self-assembled stacks of metalloporphyrins aligned at an angle with respect to the surface with interplanar distance measuring 15.04 Å for ZnTCB_4_PP, 16.03 Å for ZnTCH_4_PP, and 19.15 Å for ZnTCO_4_PP [40]. The relative intensity of the Bragg peak at 2θ = 5.5° with respect to the sharp peak at 2θ = 16.85° grows dramatically with the increase in length of linear peripheral alkyl chain length. The metalloporphyrin thin films appear to have higher molecular ordering than their previously reported free-base counterparts, as evidenced from the high signal to noise ratio. Interestingly, ZnTCEH_4_PP shows an absence of out-of-plane ordering of metalloporphyrins in thin films, contrary to the free-base study. However, the in-plane 2θ peaks are the most intense for ZnTCEH_4_PP among the four derivatives.

## 3. Discussion

UV-Vis absorption studies show that all the metalloporphyrin derivatives exhibit identical spectra in solution. The functionalization of the metalloporphyrin with peripheral alkyl groups does not alter the absorption characteristics in solution. The absence of the first and second Q bands is typical with Zn porphyrins [42,43]. When the metalloporphyrin solutions are cast to fabricate thin films, the alkyl chain length and branching influences the self-assembly of the molecules with respect to each other and to the substrate. Thus, the absorption spectra for solution-cast thin films exhibit a distinct red shift. This shift and the broadening of the Soret band are caused by the transition dipole orientation and strong excitonic coupling of the porphyrin macrocycle. ZnTCEH_4_PP, for example, has the broadest Soret band and an amplified Q_4_ feature. This could be indicative of strong π–π stacking and nematic ordering of the dipoles along the substrate. A shoulder in the Soret band for ZnTCH_4_PP, ZnTCEH_4_PP, and ZnTCO_4_PP is further indicative of strong excitonic coupling, as seen in previous studies [44]. This was evidenced in the exciton diffusion studies with all the three derivatives having higher diffusion lengths compared to ZnTCB_4_PP.

The metalloporphyrins have comparable, if not longer, average singlet emission decay lifetime in thin films than in solution. This interesting property likely arises due to the peripheral alkyl chain influenced by the self-assembly of molecules in thin films. ZnTCH_4_PP exhibits the longest emission decay lifetime, followed closely by ZnTCO_4_PP. The alkyl chains behave as molecular spacers that keep the porphyrin macrocycle apart. With a small spacer, the macrocycles are assembled close to each other which quenches the singlet emission, as seen in ZnTCB_4_PP.

The exciton diffusion simulation results were comparable with previous reports [25]. ZnTCB_4_PP has the lowest exciton diffusion length as it has the shortest lifetime decay and low quenching efficiency. ZnTCH_4_PP has the longest singlet lifetime decay but did not have long exciton diffusion lengths or high diffusion coefficient due to low quenching efficiency. The decay lifetime and quenching efficiency in ZnTCEH_4_PP were modest and, hence, have a respectable exciton diffusion length of 13.74 nm. ZnTCO_4_PP, however, has a remarkable diffusion coefficient and one of the highest diffusion lengths (80.67 nm) reported in the literature for similar molecules. Clearly, the nature of self-assembly of molecules in thin films has a high impact on exciton diffusion. Modulation of peripheral alkyl groups influences the transition dipole interaction and macrocyclic stacking. High exciton diffusion in ZnTCO_4_PP, for example, could be attributed to highly ordered molecular dipoles and long-range ordering of molecules. The evidence of this ordering was seen in powder X-ray diffraction studies.

Although all derivatives exhibit high transition dipole ordering, evidenced by peaks 14° < 2θ < 25.45°, ZnTCB_4_PP, ZnTCH_4_PP, and ZnTCO_4_PP have strong relative intensity at peak 2θ = 5.5°. This confirmed that the molecules with linear peripheral alkyl chains are preferentially stacked with porphyrin edge-on alignment [45]. The preference for out-of-plane stacking increases with the increase in the peripheral alkyl chain length. In the case of ZnTCO_4_PP, this kind of edge-on molecular arrangement is most dominant. The interplanar distance for out-of-plane arrangement increases from 15.04 Å in ZnTCB_4_PP to 19.15 Å in ZnTCO_4_PP, as seen in Table S1. This sharp Bragg peak is consistent with previously reported work on similar molecules [46,47]. The absence of peak at 2θ = 5.5°, in the case of ZnTCEH_4_PP, suggests a lack of out-of-plane crystalline arrangement of molecules, perhaps caused by the cis-trans branching in the peripheral alkyl chain [48]. This branching is likely why ZnTCEH_4_PP does not have strong quenching efficiency. Surprisingly, the 2-ethylhexyl free-base derivative exhibited a sharp Bragg peak at 2θ = 5.78° in the earlier study [19]. The absence of a Bragg peak in the metalloporphyrin could also be a result of the addition of the Zn (II) ion and the molecular shape-induced effects thereof. However, it should be noted that ZnTCEH_4_PP has the strongest in-plane molecular ordering among the four derivatives, forming a highly nematic and/or homeotropic arrangement of molecules. Similar nematic ordering is present in thin films of other linear chain derivatives as well. The identical peak intensity for ZnTCB_4_PP and ZnTCH_4_PP (14° < 2θ < 25.45°) suggest that both metalloporphyrins have similar tendency for in-plane molecular self-assembly. The low peak intensity in ZnTCO_4_PP (14° < 2θ < 25.45°) reinforces the proposition that the octyl derivative strongly prefers edge-on stacking over nematic/homeotropic.

**Figure 5:**
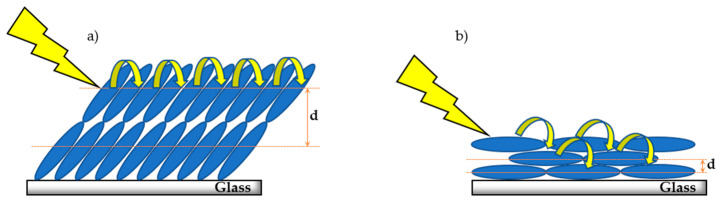
Proposed dominant molecular domain arrangement and exciton diffusion pathway in (**a**) octyl derivative—ZnTCO_4_PP and (**b**) 2-ethylhexyl derivative—ZnTCEH_4_PP of metalloporphyrin thin films.

The 29-nm red shift and broadening observed in the UV-Vis spectra of metalloporphyrin solution versus thin films is a good indication of the formation of J-aggregates [49]. This is highly desirable for rapid- and long-exciton diffusion, as it has been previously shown that H-aggregation behaves as an exciton trap and is detrimental for excited-state energy transfer [50,51]. The aggregation exists in large crystalline domains with transition dipoles oriented in different angles with respect to the substrate, as discussed earlier. We can observe how the self-assembly affects the directionality of exciton diffusion by comparing the two films with the most dramatic difference in angular orientation of the transition dipoles with respect to the substrate ((a) octyl and (b) 2-ethylhexyl derivatives), as depicted in Figure 5.

As mentioned earlier, the strong Bragg peak in the octyl derivative thin film indicates that the transition dipoles are assembled at a large tilt angle with respect to the substrate. Since the Bragg peak is the most dominant feature in the XRD pattern of the octyl derivative, it can be assumed that this tilted angle orientation is the largest domain in the thin films. The large tilt angle between the stack direction and substrate results in long interplanar distance and could lead to short stack height. This large distance and short stack height would make interplanar exciton hopping unlikely. However, given the strong excitonic coupling between the adjacent stack metalloporphyrin macrocycles, leading to enhanced π–π interaction, it is reasonable to envision that the exciton diffusion will favor a lateral pathway. The unperturbed lateral diffusion through a well-ordered domain could be one of the reasons behind the impressive exciton diffusion lengths in the octyl derivative. In the 2-ethylhexyl derivative, however, the metalloporphyrin transition dipoles are arranged parallel to the substrate. This would lead to shorter interplanar distances and higher molecular stacks. ZnTCEH_4_PP also boasts of high excitonic coupling, indicating strong π–π macrocyclic interaction. Therefore, we propose that excitons would prefer a perpendicular diffusion pathway with respect to the substrate in these films. These insights into the directionality of exciton diffusion could be very helpful when designing the architecture of an optoelectronic device. Given that the net electrical charge of an exciton is neutral, and, hence, their inoculation to applied potential, it is impressive that self-assembly can be used as a guiding tool to modulate the directionality of excitons in these materials.

This work demonstrates that peripheral alkyl chains and the metal atom in Zn porphyrins influences self-assembly of molecules in thin films. It is also evident from this work that the nature of molecular self-assembly in thin films play a crucial role in excited-state energy diffusion. This work would be useful for molecular design and engineering aiming at optoelectronic applications where efficient/rapid exciton diffusion is desired.

## 4. Materials and Methods

### 4.1. Materials and Instrumentation

Zinc acetate dihydrate, 1-bromobutane, 1-bromohexane, 3-(bromomethyl)heptane, 1-bromooctane, chloroform (CHCl_3_), dichloromethane (CH_2_Cl_2_), tetrahydrofuran (THF), chlorobenzene (CB), and methanol (CH_3_OH) were purchased from Sigma-Aldrich and used without further purification. 5,10,15,20-Tetrakis(4-carboxyphenyl)porphyrin (TCPP) was purchased from TCI America and used as received. ^1^H NMR measurements were carried out using a JEOL 300-MHz NMR and JEOL 500-MHz NMR. UV-Vis absorption spectrometric data for solution and thin films were obtained using Cary 300 UV-Vis spectrophotometer. Solid state photoluminescence measurements were carried out on Jobin Yvon-Spex Fluorolog. PL decay lifetimes were obtained using a diode laser with a repetition rate of 1 MHz and excitation wavelengths of 389 nm. XRD θ/2θ patterns were obtained using a Panalytical X’Pert Pro MPD with Ni filtered Cu K alpha radiation (*λ* = 1.541 Å) at 45 kV, 40 mA. The experiments were performed from 3° to 120° with 0.02° step size and a counting time of 0.35 s per point.

### 4.2. Preparation of Porphyrin Derivatives

The following free-base 5,10,15,20-tetrakis(4-carboxyphenyl)porphyrin (TCA_4_PP) were synthesized using previously reported synthetic methods [19].

5,10,15,20-tetrakis[4-(butoxycarbonyl)phenyl]porphyrin (TCB_4_PP), 5,10,15,20-tetrakis[4-(hexyloxycarbonyl)phenyl]porphyrin (TCH_4_PP), 5,10,15,20-tetrakis[4-((2-ethylhexyl)oxycarbonyl)phenyl]porphyrin (TCEH_4_PP) and 5,10,15,20-tetrakis[4-(octyloxycarbonyl)phenyl]porphyrin (TCO_4_PP).

**General Procedure for Zn Porphyrin Synthesis**

Zn-5,10,15,20-tetrakis(4-carboxyphenyl)porphyrin (Zn-TCA_4_PP): 5,10,15,20-tetrakis(4-carboxyphenyl)porphyrin (50 mg, 0.074 mmol) was reacted with an excess of Zn(OAc)_2_·2H_2_O (15 mg, 0.11 mmol) in CH_2_Cl_2_:CH_3_OH (20 mL, 10:1) at 65 °C for 2 h in a dark environment. The reaction mixture was rinsed with Milli-Q. The product was purified by silica gel column chromatography with CHCl_3_:(CH_2_)_4_O (20:1) as mobile phase. Purity was confirmed with TLC, ^1^H NMR, and MALDI-TOF mass spectrometry.

Zn-5,10,15,20-tetrakis[4-(butoxycarbonyl)phenyl]porphyrin (Zn-TCB_4_PP):

Column chromatography using CHCl_3_:(CH_2_)_4_O (20:1) afforded 0.098 g (96%) of a slight reddish-purple solid. ^1^H NMR (300 MHz, CDCl_3_, TMS, δ): 8.86 (s, 8H), 8.35 (d, *J* = 8.0 Hz, 8H), 8.23 (d, *J* = 8.3 Hz, 8H), 4.40 (t, *J* = 6.5 Hz, 8H), 1.83 (pnt, *J* = 7.0 Hz, 8H), 1.56 (sxt, *J* = 7.4 Hz, 8H), and 1.01 (t, *J* = 7.3 Hz, 12H). UV-Vis *λ*_max_ (CHCl_3_, ε = M^−1^cm^−1^): 421 nm (ε = 935,320), 549 nm (ε = 38,651), and 587 nm (ε = 7558.9). MALDI-TOF (Calcd for C_64_H_61_N_4_O_8_Zn, [M + H]^+^): 1077.37, found: M = 1077.10.

Zn-5,10,15,20-tetrakis[4-(hexyloxycarbonyl)phenyl]porphyrin (Zn-TCH_4_PP):

Column chromatography using CHCl_3_:(CH_2_)_4_O (20:1) afforded 0.08 g (71%) of a slight reddish-purple solid. ^1^H NMR (300 MHz, CDCl_3_, TMS, δ): 8.92 (s, 8H), 8.45 (d, *J* = 8.0 Hz, 8H), 8.31 (d, *J* = 8.0 Hz, 8H), 4.53 (t, *J* = 6.6 Hz, 8H), 1.92 (pnt, *J* = 7.04 Hz, 8H), 1.59 (m, 8H), 1.43 (m, 16H), and 0.96 (t, *J* = 7.0 Hz, 12H). UV-Vis *λ*_max_ (CHCl_3_, ε = M^−1^ cm^−1^): 421 nm (ε = 758,500), 548 nm (ε = 29,000), and 583 nm (ε = 13,000). MALDI-TOF (Calcd for C_72_H_76_N_4_O_8_Zn): 1188.50, found: M = 1188.06.

Zn-5,10,15,20-tetrakis[4-((2-ethylhexyl)oxycarbonyl)phenyl]porphyrin (Zn-TCEH_4_PP):

Column chromatography using CHCl_3_:(CH_2_)_4_O (20:1) afforded 0.08 g (64%) of a slight reddish-purple solid. ^1^H NMR (300 MHz, CDCl_3_, TMS, δ): 8.93 (s, 8H), 8.44 (d, *J* = 8.0 Hz, 8H), 8.30 (d, *J* = 8.3 Hz, 8H), 4.44 (t, *J* = 6.5 Hz, 8H), 1.87 (pnt, *J* = 7.0 Hz, 8H), 1.60 (m, *J* = 7.4 Hz, 10H), 1.43 (m, *J* = 7.4 Hz, 20H), 1.05 (t, *J* = 7.3 Hz, 12H), and 0.96 (t, *J* = 7.3 Hz, 12H). UV-Vis *λ*_max_ (CHCl_3_, ε = M^−1^ cm^−1^): 426 nm (ε = 653,000), 553 nm (ε = 29,000), and 596 nm (ε = 13,000). MALDI-TOF (Calcd for C_80_H_93_N_4_O_8_Zn, [M + H]^+^): 1301.62, found: M = 1301.33.

Zn-5,10,15,20-tetrakis[4-(octyloxycarbonyl)phenyl]porphyrin (Zn-TCO_4_PP):

Column chromatography using CHCl_3_:(CH_2_)_4_O (20:1) afforded 0.08 g (66%) of a slight reddish-purple solid. ^1^H NMR (300 MHz, CDCl_3_, TMS, δ): 8.87 (s, 8H), 8.36 (d, *J* = 8.0 Hz, 8H), 8.24 (d, *J* = 8.3 Hz, 8H), 4.42 (t, *J* = 6.5 Hz, 8H), 1.85 (pnt, *J* = 7.0 Hz, 8H), 1.43 (m, *J* = 7.4 Hz, 40H), and 0.83 (t, *J* = 7.3 Hz, 12H). UV-Vis *λ*_max_ (CHCl_3_, ε = M^−1^ cm^−1^): 421 nm (ε = 453,000), 549 nm (ε = 21,000), and 584 nm (ε = 9000). MALDI-TOF (Calcd for C_80_H_91_N_4_O_8_Zn, [M − H]^−^): 1299.62, found: M = 1299.76.

### 4.3. Zn-Porphyrin Thin-Film Preparation and Characterization

Glass microscope slides were sonicated with glass cleaning detergent and isopropyl alcohol. They were dried with N_2_ gas followed by UV–ozone treatment. Zn-(Alkyloxycarbonyl)phenylporphyrin solutions of 6-mM strength were made in chlorobenzene under N_2_ atmosphere and stirred for 24 h. Dopant solution was made by dissolving C_60_ in chlorobenzene and stirring it for 24 h. Prior to spin-casting, glass slides were warmed for 20 min at 70 °C to encourage uniform film formation. The thin films were fabricated by casting the Zn porphyrin solutions at 2000 rpm for 60 s under N_2_ atmosphere. The C_60_ volume fraction (volume occupied by C_60_ molecules versus blend volume) of 0.06% and 0.2% were calculated using previously reported methods [24,31]. The Zn porphyrin densities were experimentally determined to be ~0.97 g cm^−3^. Emission decay lifetimes of thin films were measured using a laser repetition rate of 1 MHz with excitation at 389 nm. PL(t) data were fitted to triple exponential decays using Igor Pro 6.3 software. The PL decays, and steady-state quenching efficiencies and *v*_frac_ were used to obtain exciton diffusion coefficients and exciton diffusion lengths using Monte Carlo diffusion software simulation [31].

## 5. Conclusions

We synthesized four (alkyloxycarbonyl)phenyl derivatives of Zn metalloporphyrins with varying alkyl chain lengths. It was found that the peripheral alkyl chain substituents and the Zn (II) ion in the porphyrin macrocycle influence the self-assembly arrangements of molecules in thin films. These molecular arrangements were studied using UV-Vis absorption spectroscopy and X-ray diffractometry. The effects of these arrangements were ascertained by obtaining average singlet emission lifetime decays using time-correlated single-photon counting. The thin films were doped with C_60_ quencher molecules in volume fractions of 0.06% and 0.2% to obtain relative quenching efficiency. The transition dipole orientation, with respect to the substrate and neighboring transition dipoles, has an impact on the exciton diffusion in thin films. To establish diffusion coefficient and diffusion lengths, relative quenching efficiency, PL decay, and dopant volume fractions were employed in a Monte Carlo simulation as input. The exciton diffusion lengths for ZnTCB_4_PP, ZnTCH_4_PP, and ZnTCEH_4_PP were found to be consistent with previously reported lengths in the literature. ZnTCO_4_PP outshone all the other derivatives with an astounding exciton diffusion length of 80.67 nm. A comparison of exciton diffusion in ZnTCO_4_PP and ZnTCEH_4_PP thin films was discussed where it was posited that excitons diffuse laterally in the octyl derivative as opposed to perpendicularly with respect to the substrate in the branched 2-ethylhexyl derivative. This work demonstrates that modulation of peripheral alkyl chain lengths and inserting a metal atom in the porphyrin macrocycle core can influence self-assembly of molecules in thin films. The resultant molecular orientation and arrangement affects the electronic, structural, and photophysical properties of the materials. Molecular self-assembly can be used as a tool to modulate and direct exciton diffusion in organic thin films engineered for optoelectronic and photonic applications.

## Figures and Tables

**Table 1:** Alkylated ZnTCA_4_PP summary of average singlet lifetime decays (*τ*_S1_), relative steady state quenching efficiency (*Q*) for dopant volume fractions 0.06% and 0.2%, exciton diffusion coefficient (*D*) and diffusion length (*L*_D_).

Material	*τ*_S1_ (ns)	*Q*_0.06%_	*D* × 10^−4^ (cm^2^/s)	*L*_D_ (nm)	*Q*_0.2%_	*L*_D_ (nm)
ZnTCB_4_PP	1.52 ± 0.07	0.37	1.0 ± 0.3	9.42 ± 1.2	0.77	8.93 ± 0.4
ZnTCH_4_PP	2.30 ± 0.11	0.37	0.90 ± 0.2	11.16 ± 1.2	0.78	9.31 ± 0.4
ZnTCEH_4_PP	1.88 ± 0.04	0.46	1.68 ± 0.2	13.74 ± 0.7	0.73	7.38 ± 0.1
ZnTCO_4_PP	2.02 ± 0.05	0.89	50.20 ± 4.1	77.98 ± 3.0	0.95	28.16 ± 0.8

## Data Availability

The data presented in this study are available in supplementary material.

## Supplementary Materials

The following are available online. Figure S1: Time-resolved fluorescence spectra and first order fitting of (a) ZnTCB_4_PP, (b) ZnTCH_4_PP, (c) ZnTCEH_4_PP and (d) ZnTCO_4_PP. Figure S2: UV-Vis absorption spectra of spin-cast metalloporphyrins thin films with and without C_60_. Table S1: XRD Diffraction Data for ZnTCB_4_PP, ZnTCH_4_PP, ZnTCEH_4_PP and ZnTCO_4_PP (Cu Kα radiation of *λ* = 1.541 Å). Figures S3–S10: ^1^H-NMR and MALDI-TOF Mass Spectra for ZnTCB_4_PP, ZnTCH_4_PP, ZnTCEH_4_PP and ZnTCO_4_PP.

## Sample Availability

Samples of the compounds ZnTCB_4_PP, ZnTCH_4_PP, ZnTCEH_4_PP and ZnTCO_4_PP are available from the authors.
