# Supplementary material for: Self-Assembly-Directed Exciton Diffusion in Solution-Processable Metalloporphyrin Thin Films
Source: Molecules. 2021 Dec 22;27(1):35. doi: 10.3390/molecules27010035 (PMC8746414; doi:10.3390/molecules27010035)

Supporting Information

Self-Assembly-Directed Exciton Diffusion in Solution-Processable Metalloporphyrin Thin Films

Abhishek Shibu, Camilla Middleton, Carly O. Kwiatkowski, Meesha Kaushal, Jonathan H. Gillen and Michael G. Walter*

[*] Prof. M. G. Walter, Corresponding Author

Department of Chemistry

University of North Carolina at Charlotte

Charlotte, NC 28223 (USA)

Figure S1. Time-resolved fluorescence spectra and first order fitting of a) $ZnTCB_4PP$, b) $ZnTCH_4PP$, c) $ZnTCEH_4PP$ and d) $ZnTCO_4PP$.

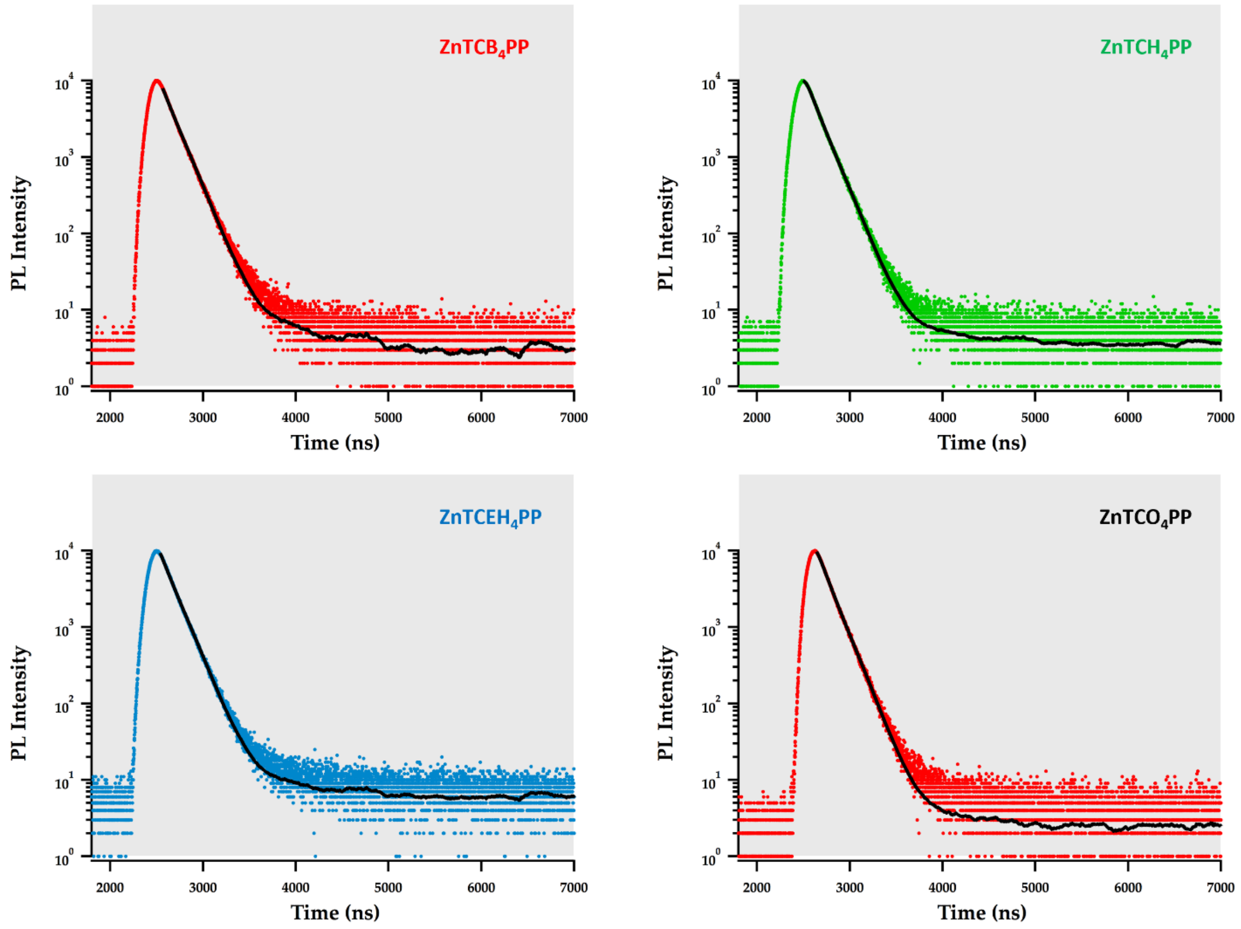

Metalloporphyrin	τ_{S1} (ns)
ZnTCB$_4$PP	1.89
ZnTCH$_4$PP	1.89
ZnTCEH$_4$PP	1.89
ZnTCO$_4$PP	1.85

Figure S2: UV-Vis absorption spectra of spin-cast metalloporphyrins thin films of (a)ZnTCB$_4$PP, (b) ZnTCH$_4$PP, (c) ZnTCEH$_4$PP and (d) ZnTCO$_4$PP. Each plot includes spectra of pristine films (-), films doped with v_{frac} 0.06% (-) and films doped with v_{frac} 0.2% (-)

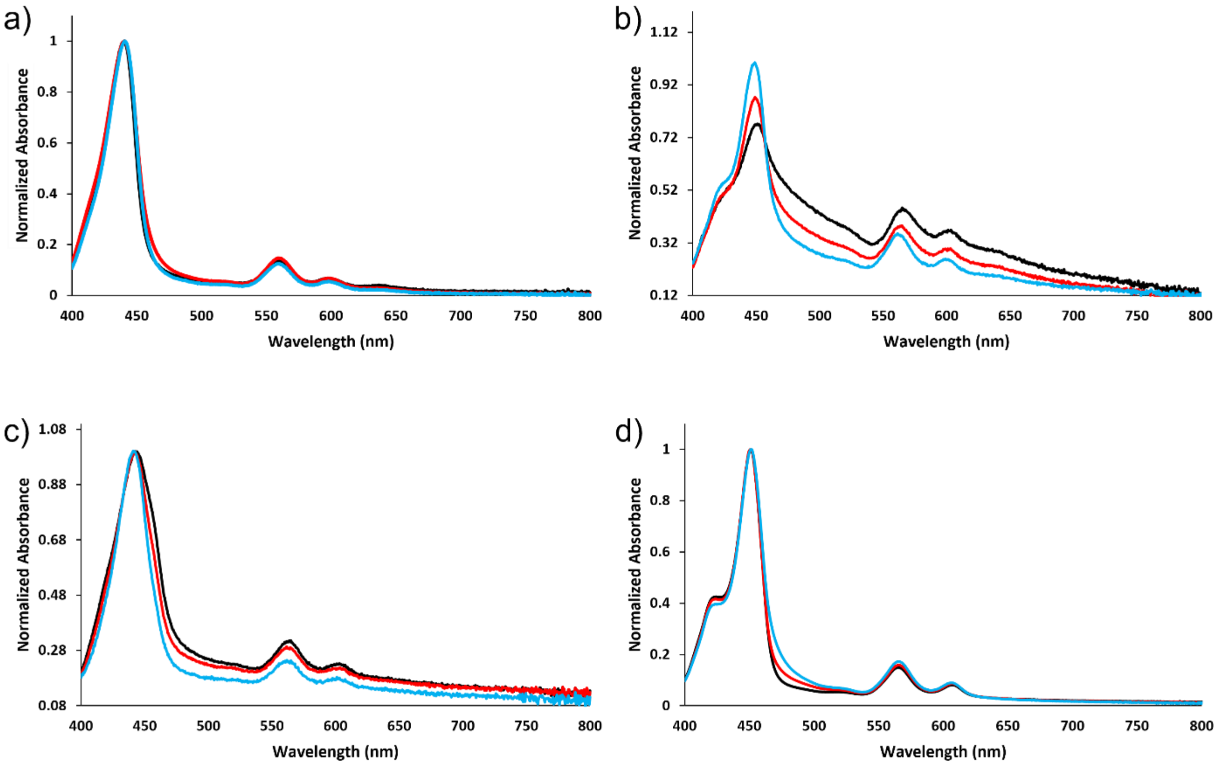

Table S1: XRD Diffraction Data for $ZnTCB_4PP$, $ZnTCH_4PP$, $ZnTCEH_4PP$ and $ZnTCO_4PP$ (Cu Kα radiation of λ = 1.541 Å).

Material	Peak (2θ)	d-spacing (Å)	Intensity	Rel Intensity
**$ZnTCB_4PP$**	5.87	15.05	77	0.31
	13.95	6.34	122	0.49
	16.81	5.27	250	1.00
	18.73	4.74	62	0.25
	25.45	3.50	57	0.23
**$ZnTCH_4PP$**	5.51	16.03	155	0.58
	14.09	6.28	114	0.43
	16.91	5.24	265	1.00
	18.43	4.81	64	0.24
	25.35	3.51	57	0.22
**$ZnTCO_4PP$**	4.61	19.16	365	2.97
	9.21	9.60	30	0.24
	13.87	6.38	64	0.52
	16.87	5.25	123	1.00
	18.45	4.81	52	0.42
	25.33	3.51	49	0.40
**$ZnTCEH_4PP$**	14.07	6.29	188	0.40
	16.85	5.26	474	1.00
	18.47	4.80	87	0.18
	25.57	3.48	77	0.16

Figure S3: ^{1}H-NMR of $ZnTCB_4PP$

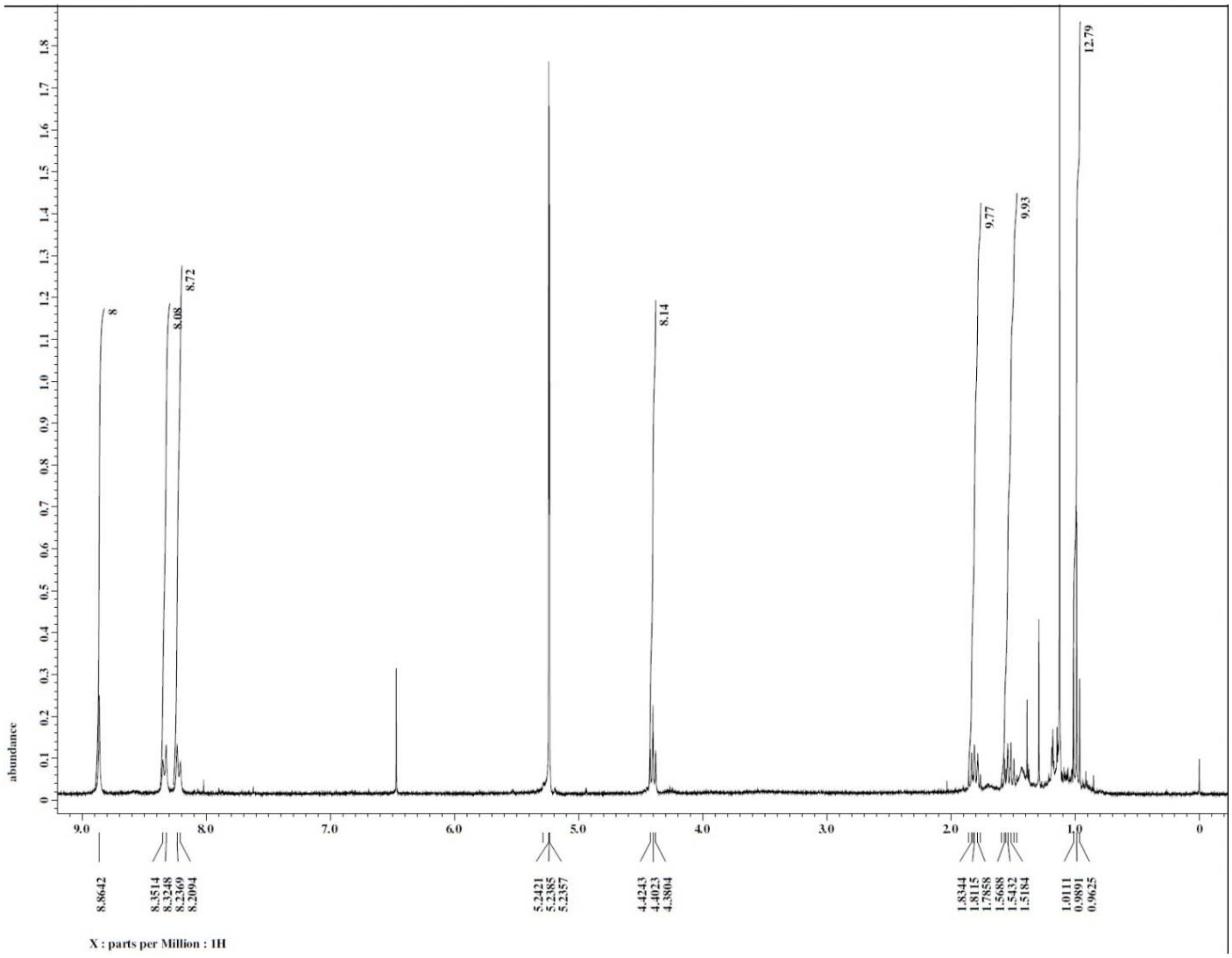

Figure S4: MALDI-TOF Mass Spectra of $ZnTCB_4PP$

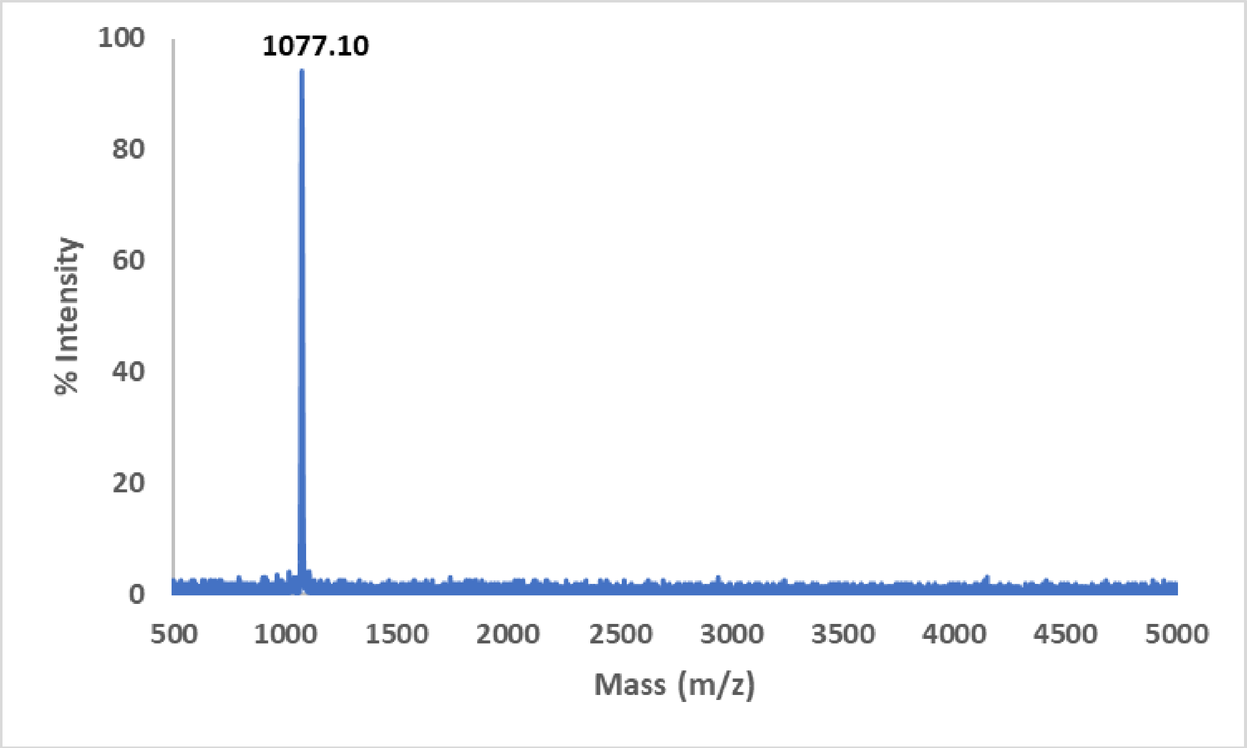

Figure S5: ^{1}H-NMR of $ZnTCH_4PP$

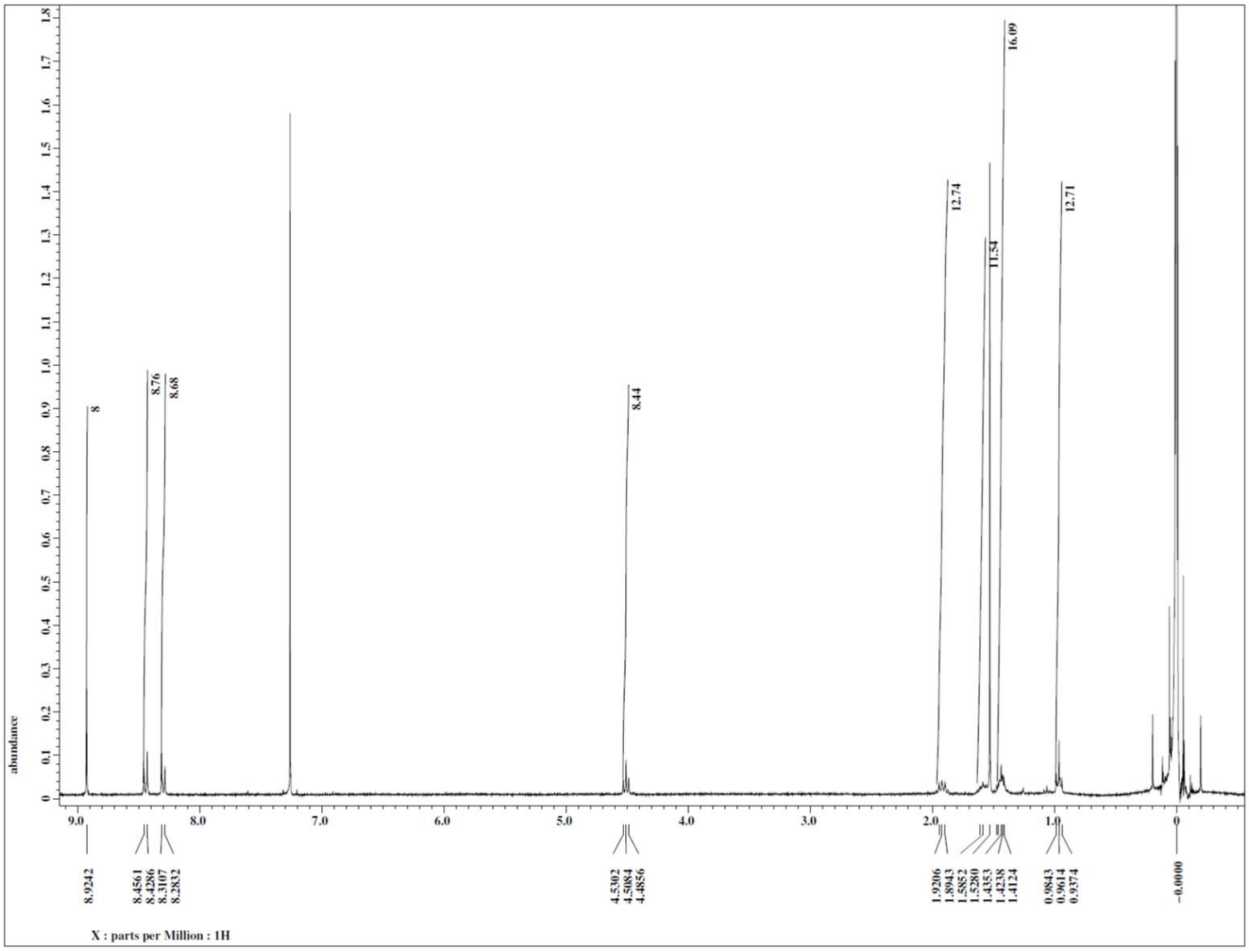

Figure S6: MALDI-TOF Spectra of $ZnTCH_4PP$

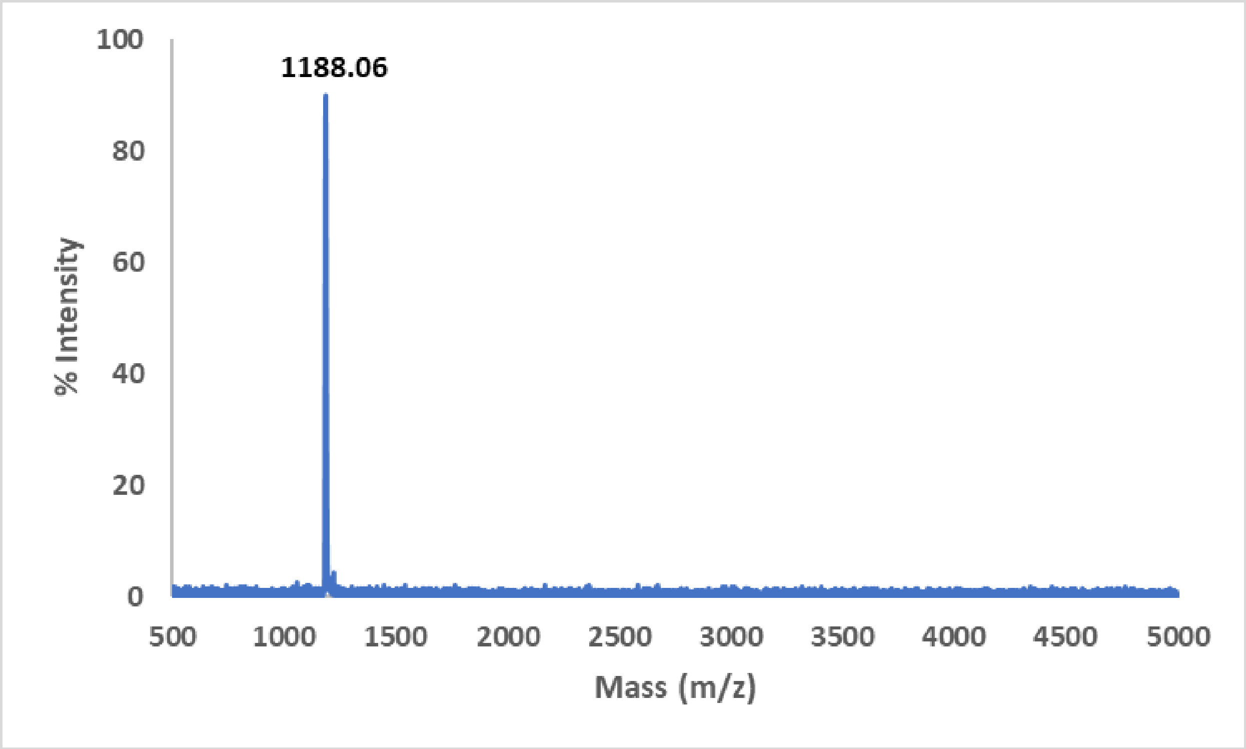

Figure S7: ^{1}H-NMR of $ZnTCEH_4PP$

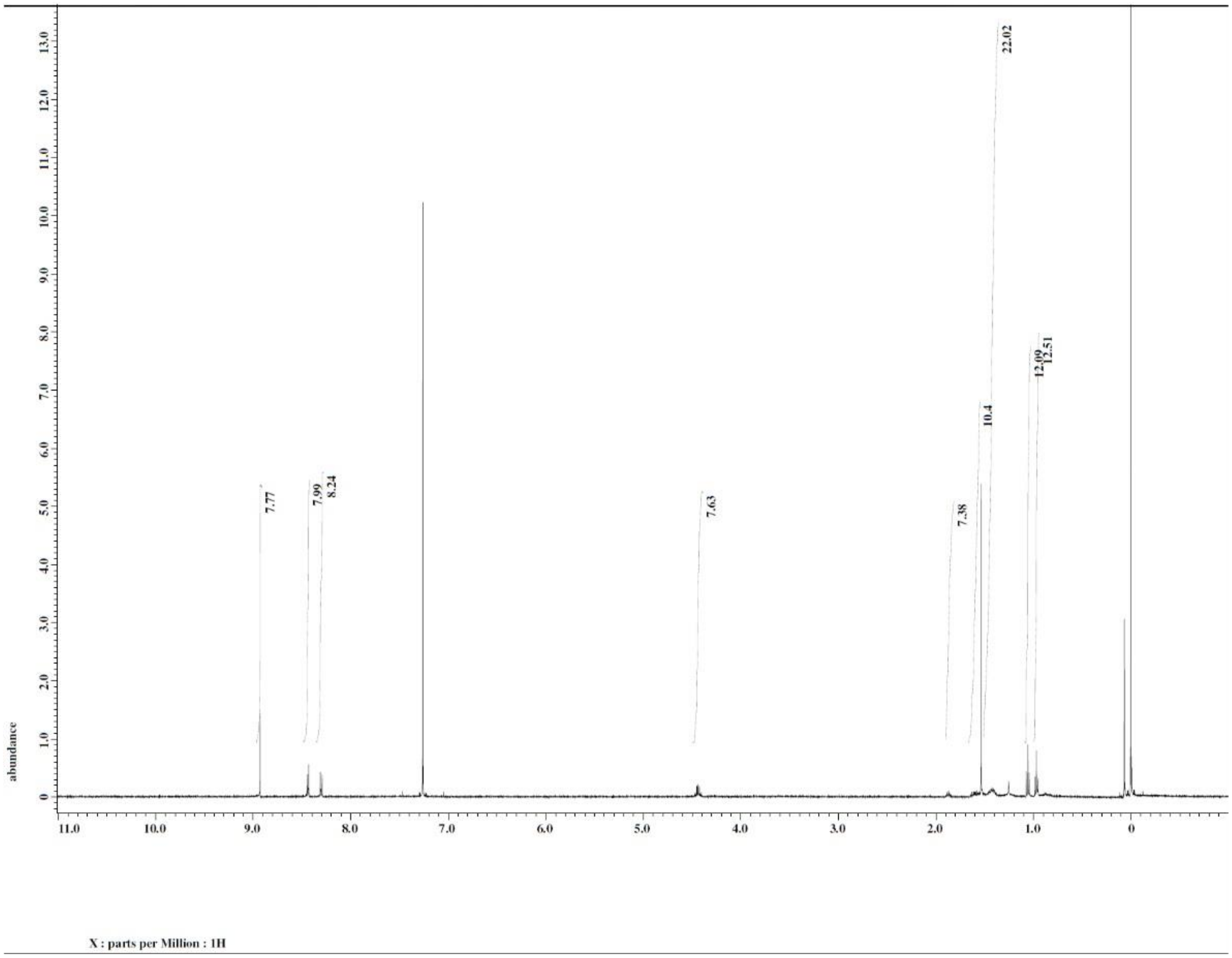

Figure S8: MALDI-TOF Mass Spectra of $ZnTCEH_4PP$

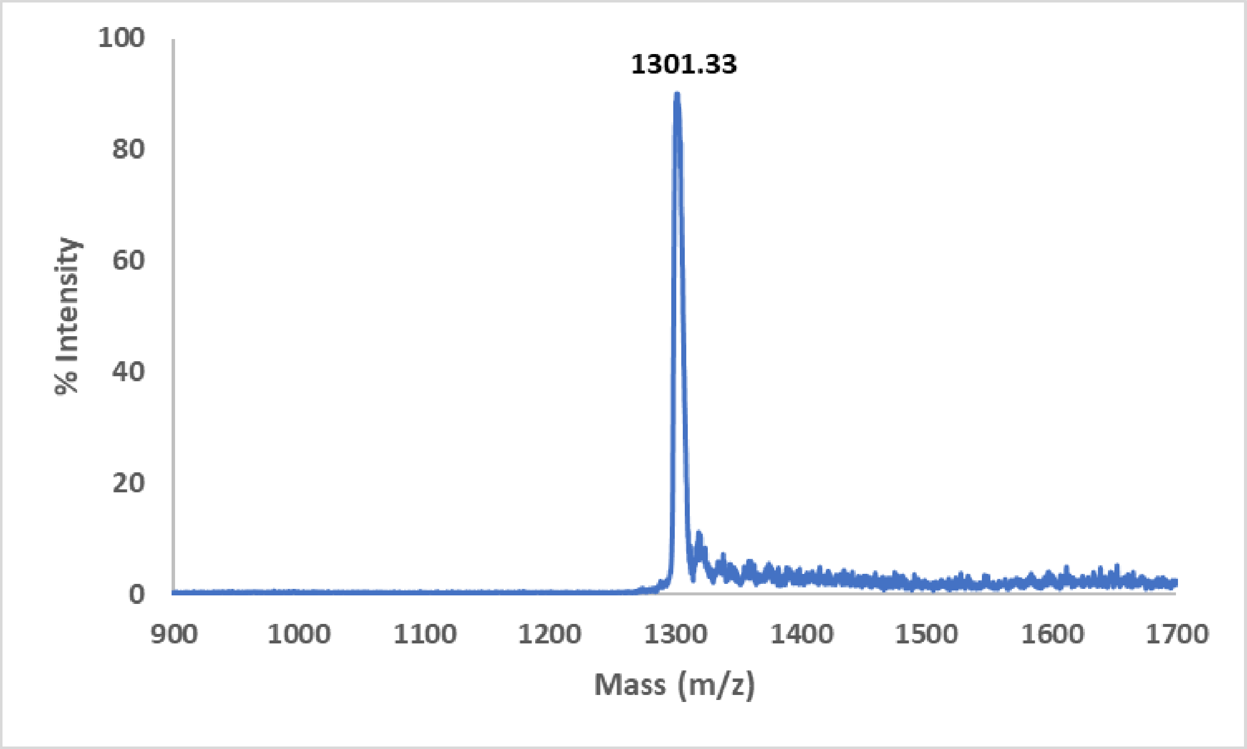

Figure S9: ^1H-NMR of $ZnTCO_4PP$

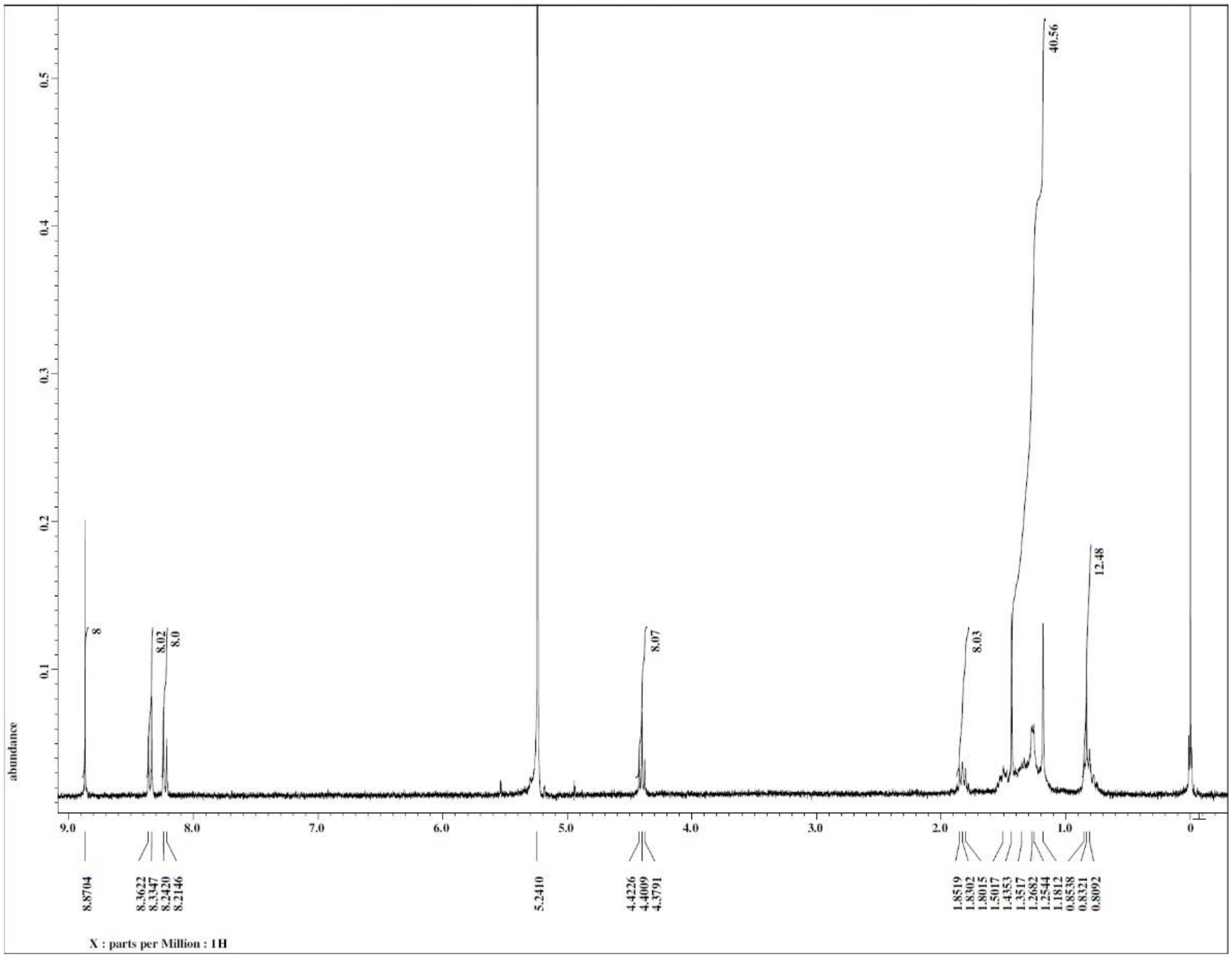

Figure S10: MALDI-TOF Mass Spectra of $ZnTCO_4PP$

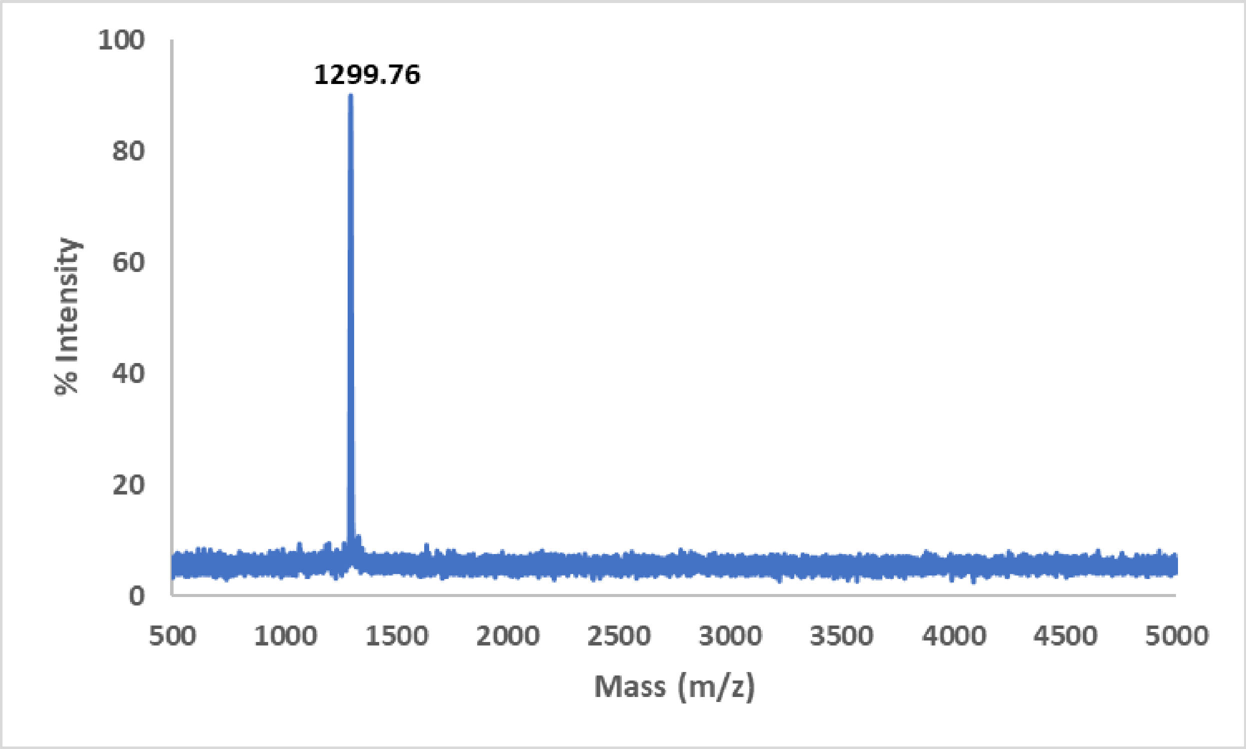

Supplement: Supplementary file 1 [file molecules-27-00035-s001.zip › molecules-1484662-supplementary.pdf]
